# High expression of HNRNPR in ESCA combined with ^18^F-FDG PET/CT metabolic parameters are novel biomarkers for preoperative diagnosis of ESCA

**DOI:** 10.1186/s12967-022-03665-4

**Published:** 2022-10-04

**Authors:** Xiao-Yu Liu, Yan Gao, Xue-Yan Kui, Xu-Sheng Liu, Yao-hua Zhang, Yu Zhang, Chang-Bin Ke, Zhi-Jun Pei

**Affiliations:** 1 Department of Nuclear Medicine and Institute of Anesthesiology and Pain, Taihe Hospital, Hubei University of Medicine, Shiyan, Hubei China; 2 Hubei Key Laboratory of Embryonic Stem Cell Research, Shiyan, Hubei China; 3 Taihe Hospital, Postgraduate Training Basement of Jinzhou Medical University, Hubei University of Medicine, Shiyan, Hubei China

**Keywords:** Esophageal carcinoma (ESCA), PET/CT, Heterogeneous nuclear ribonucleoprotein R (HNRNPR), m6A modification, Glycolysis

## Abstract

**Background:**

The aim of this study was to determine the expression and function of heterogeneous nuclear ribonucleoprotein R (HNRNPR) in esophageal carcinoma (ESCA), the correlation between its expression and ^18^F-fluorodeoxyglucose (^18^F-FDG) positron emission tomography/computerized tomography scan (PET/CT)-related parameters. We also investigated whether ^18^F-FDG PET/CT can be used to predict the expression of HNRNPR in ESCA.

**Methods:**

We analyzed patients with ESCA who underwent ^18^F-FDG PET/CT before surgery, and their tissues were stained with HNRNPR IHC. The associated parameters were derived using the ^18^F-FDG PET imaging data, and the correlation with the IHC score was evaluated. The Oncomine, TCGA, and GEO datasets were used to investigate HNRNPR expression in the pan- and esophageal cancers, as well as its relationship with N6-methyladenosine (m6A) modification and glycolysis. The R software, LinkedOmics, GeneMANIA, and StringOnline tools were used to perform GO/KEGG, GGI, and PPI analyses on the HNRNPR.

**Results:**

HNRNPR is highly expressed in the majority of pan-cancers, including ESCA, and is associated with BMI, weight, and history of reflux in patients with ESCA. HNRNPR is somewhat accurate in predicting the clinical prognosis of ESCA. HNRNPR expression was positively correlated with SUV_max_, SUV_mean_, and TLG in ESCA (p < 0.05). The combination of these three variables provides a strong predictive value for HNRNPR expression in ESCA. GO/KEGG analysis showed that HNRNPR played a role in the regulation of cell cycle, DNA replication, and the Fannie anemia pathway. The analysis of the TCGA and GEO data sets revealed a significant correlation between HNRNPR expression and m6A and glycolysis-related genes. GSEA analysis revealed that HNRNPR was involved in various m6A and glycolysis related-pathways.

**Conclusion:**

HNRNPR overexpression correlates with ^18^F-FDG uptake in ESCA and may be involved in the regulation of the cell cycle, m6A modification, and cell glycolysis. ^18^F-FDG PET/CT-related parameters can predict the diagnostic accuracy of HNRNPR expression in ESCA.

## Background

According to the Global Cancer Statistics 2020, ESCA was ranked the seventh most common type of cancer (604,000 new cases) and the sixth major cause of cancer-related deaths (544,000 deaths) [1]. These figures were higher compared with those reported in 2018. Squamous cell carcinoma (SCC) and adenocarcinoma (AC) are the two pathological subtypes of ESCA, with SCC accounting for more than 90% of cases [2]. Despite the advancement of multimodality therapies such as surgery, radiotherapy, chemotherapy, and immunotherapy, patients with these cancers have a poor prognosis because most of them are diagnosed at late stage. Moreover, the mortality rates of the locally advanced ESCC are high [3–6]. Therefore, strategies that promote early diagnosis and treatment of ESCA should be developed.

Positron emission tomography/computed tomography (PET/CT) with ^18^F-fluorodeoxyglucose (^18^F-FDG) is a non-invasive, efficient, and clinically useful technique [7, 8]. This technique can be used to evaluate glucose metabolism and the burden of tumor cells in vivo by monitoring the uptake of FDG, a glucose analogue [9]. Both glucose intake and aerobic glycolysis are increased in tumor cells as a result of the Warburg effect, which may be associated with rapid cell proliferation, invasion, and migration in tumors [10–12]. Additionally, up-regulation of glycolysis results in increased FDG uptake. Numerous studies now demonstrate that ^18^F-FDG-PET can accurately predict pathologic response and outcomes in patients with locally advanced ESCA [13–15]. Furthermore, FDG-PET scans are routinely used to stage esophageal and EGJ tumors, and PET scan parameters such as the maximum standardized uptake value (SUV_max_) have been demonstrated to be potential predictors of patient prognosis [7, 16]. While PET has been shown to be a useful tool for detecting ESCA, there are currently no excellent biomarkers for its diagnosis.

HNRNPR is an RNA binding protein that belongs to the hnRNPs (heterogeneous nuclear ribonucleoproteins) family [17]. Previous studies have demonstrated that the level of expression of this family varies across cancers, implying that they play an important role in tumorigenesis. HNRNPR has recently shifted its focus to the mechanism of action in the nervous system [18, 19]. Although it has been established as a proto-oncogene associated with gastric cancer (GC) [20], few studies have examined its role in other cancers. There is currently no evidence that HNRNPR plays a role in ESCA.

HNRNPR and its co-family genes are highly correlated with many aspects of tumor therapy, such as m6A modification and targeted glycolytic pathway. These have been used in the management of many diseases including ESCA. However, few studies have analyzed the role of HNRNPR in ESCA.

This study mainly discusses the expression and role of HNRNPR in ESCA, as well as explores its potential as a biomarker of ESCA and association with PET-related parameters. This will provide ideas for developing an effective method for early screening of ESCA and promote its treatment.

## Materials and methods

### Expression of HNRNPR in difference datasets

We analyzed the expression of HNRNPR in different tumors in the Oncomine (www.oncomine.org) online database. The HNRNPR expression levels between cancer and normal groups were compared using the Student’s t test. The expression of HNRNPR in various pan-cancers and ESCA was determined in the TCAG database. The difference in HNRNPR expression between ESCC and normal esophageal epithelium or normal adjacent tissues were compared using the GSE45670 and GSE20347 datasets.

We analyzed the ESCA datasets from the TCAG database to determine the relationship between HNRNPR expression levels and clinical manifestations of ESCA patients, and constructed a receiver operating characteristic (ROC) curve to determine the diagnostic prediction accuracy of HNRNPR in ESCA patients. The Kaplan–Meier Plotter (http://kmplot.com/analysis/) was used to evaluate the effect of gene expression on the survival of ESCA patients.

### Immunohistochemistry (IHC)

The IHC staining assay was performed on paraffin-embedded 4-μm-thick tissue sections. After dewaxing, fixed sections were microwaved for 10 min in Tris–EDTA (pH 9. 0) (ab93684; Abcam, USA). Normal serum was sealed at room temperature for 30 min. The sections were incubated with HNRNPR rabbit polyclonal antibody (1:50, 15018-AP, Proteintech, USA) at 4 °C. The next day, the slices were rewarmed and incubated with secondary antibody HRP-labelled anti-rabbit (1:500; ab6802; Abcam, USA) antibodies at room temperature for 1 h. The sections were then stained with the Diaminobenzaldehyde (DAB) reagent.

The expression of HNRNPR was evaluated using ImageJ software. A negative result was given a score of 0, whereas low positive, positive, or high positive were scored as 1, 2, and 3, respectively. At least five fields were selected for each slice and an average value was calculated.

### Quantitative RT-PCR (qRT-PCR)

The RNA was isolated from cancerous and para-cancerous tissues using the Trizol reagent (Ambion, USA). PrimeScript™ RT Master Mix (TakaRa, Japan) was used for real-time polymerase chain reaction (RT-PCR). SYBR Green Real-time PCR Master Mix (Takara, Japan) was used to quantify mRNA expression. The 2^−ΔΔCT^ formula was used to calculate the relative expression multiple of candidate genes in unpaired samples, whereas the HNRNPR/ACTH was employed to calculate the expression of paired samples. (HNRNPR: forward primer (5′-3′), GGAGGCAAGAGAAAGGCAGATGG, and reverse primer (5′-3′), GCTGAGCGATGGGTTGGGAAC. ACTB: forward primer (5′-3′), GCACAGAGCCTCGCCTT, and reverse primer (5′-3′), GTTGTCGACGACGAGCG.)

### ^18^F-FDG PET/CT imaging and analysis of related parameters

PET/CT images of ESCA in patients who fasted for 6 h were obtained 50–60 min after ^18^F-FDG (3.7 MBq/kg) injection, as described previously [5, 21], using a 64-detector PET/CT scanner (Biograph mCT-S, Siemens, USA). After iteratively reconstructing PET images and computing semi-quantitative parameters, regions of interest (ROIs) were drawn around the tumors. The SUV_max_, mean standardised uptake value (SUV_mean_), total lesion glycolysis (TLG), and metabolic tumour volume (MTV) of each tumor were automatically calculated and recorded.

### Gene set enrichment analysis (GSEA) and bioinformatics analysis

To determine whether HNRNPR is involved in the biological processes of ESCA, we used GSEA to evaluate the gene map and associated gene correlation information for HNRNPR samples from the TCGA ESCA data set. The reference gene set was set to c2. cp. v7. 2. symbols. gmt [Curated]. Data with the FDR (q-value) < 0. 25 and P < 0. 05 were considered statistically significant.

### Enrichment analysis of HNRNPR gene co-expression network in ESCA

We investigated the co-expressed genes associated with HNRNPR expression using the stat packet of R software to analyze the TCGA ESCA datasets using Pearson’s correlation coefficient to evaluate for statistical correlation. Volcano plot and heat map were drawn using the ggplot2 package in R software. The Gene ontology (GO) and Kyoto Encyclopedia of Genes and Genomes (KEGG) pathway enrichment analysis was conducted followed by a visual analysis of the data.

### Gene–gene interaction and protein–protein interaction (GGI&PPI)

We used the GeneMANIA database (http://genemania.org) to generate gene lists and build interactive network maps to analyze the HNRNPR function and genomes. When calculating and analyzing the PPI of HNRNPR using the STRING database (www.string-db.org), we selected the minimum required interaction score as the highest confidence level (0. 900).

### Correlations between HNRNPR Expression and m6A in ESCA

There were 20 m6A-related genes used to examine the correlation between HNRNPR expression in the TCGA database and the GSE69925 data set, as well as the differential expression between groups with high and low HNRNPR expression [22, 23]. Data were analyzed using the Ggplot2 software package.

### Correlations between HNRNPR expression and glycolysis in ESCA

The correlation between HNRNPR expression and expression of glycolysis-related genes was analyzed in the TCGA database and the GSE69925 data set to further investigate the role of HNRNPR in glycolysis [24, 25].

## Result

### Differences in HNRNPR mRNA expression in pan-cancer

**Fig. 1:**
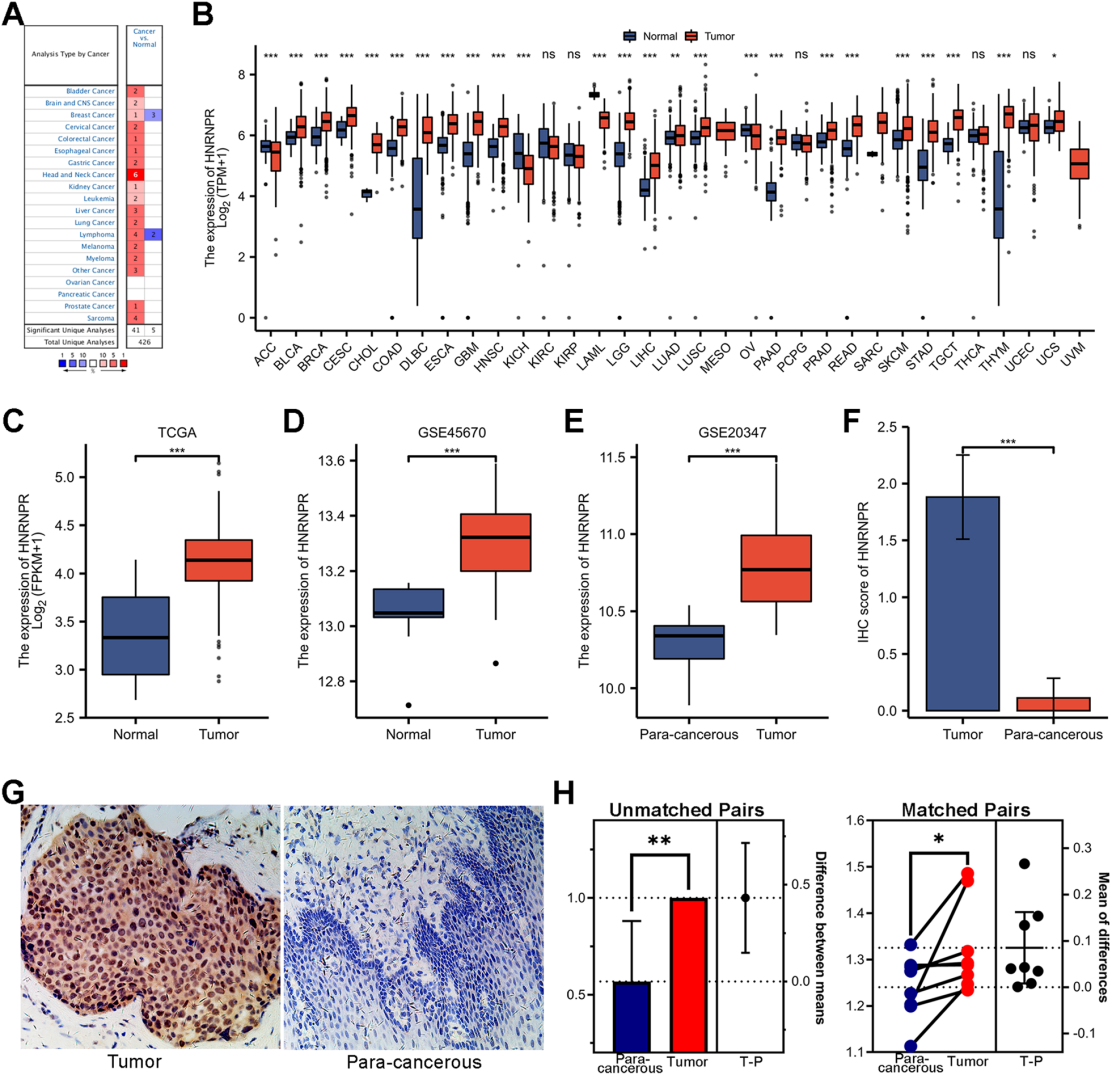
HNRNPR overexpression in ESCA and pan-cancer. **A** HNRNPR mRNA was expressed in various pan-cancers. **B** The expression level of HNRNPR mRNA was evaluated by analyzing TCGA data in various pan-cancers. **C**, **D** The difference in HNRNPR expression between ESCA cancer tissue and normal tissue in the TCGA and GSE45670 datasets. **E** The difference in HNRNPR expression between ESCA cancer and para-cancerous tissues in the GSE20347 dataset. **G**, **F** The HNRNPR expression and score in cancer and para-cancerous tissues of patients with ESCA were detected by IHC. **H** The difference in HNRNPR mRNA expression between cancer and para-cancerous tissues of ESCA patients based on qPCR test. *P < 0.05, **P < 0.01, ***P < 0.001

We analyzed the transcription level of HNRNPR in various pan-cancer and normal individuals using the Oncomine online database and TCGA dataset. Data from the Oncomine database with fold change > 1. 5 and a p-value < 0. 001 were analyzed. Results showed that HNRNPR expression level was higher level in many cancers than in normal tissue (Fig. 1A).

Further analysis revealed that the mRNA level of HNRNPR was increased in many cancers, including ESCA (Fig. 1B).

### HNRNPR expression predicted poor survival in ESCA patients

The analysis of ESCA patient data from the TCGA and GEO database revealed that HNRNPR expression was significantly elevated in cancer tissue than in normal tissue or para-cancerous tissue (Fig. 1C–E).

Next, IHC and qPCR experiments were conducted to confirm the expression and prediction accuracy of HNRNPR in ESCA. The results indicated that HNRNPR mRNA and protein level was significantly higher in para-cancerous tissues than in normal tissues, particularly in the IHC test (Fig. 1F–H).

**Fig. 2:**
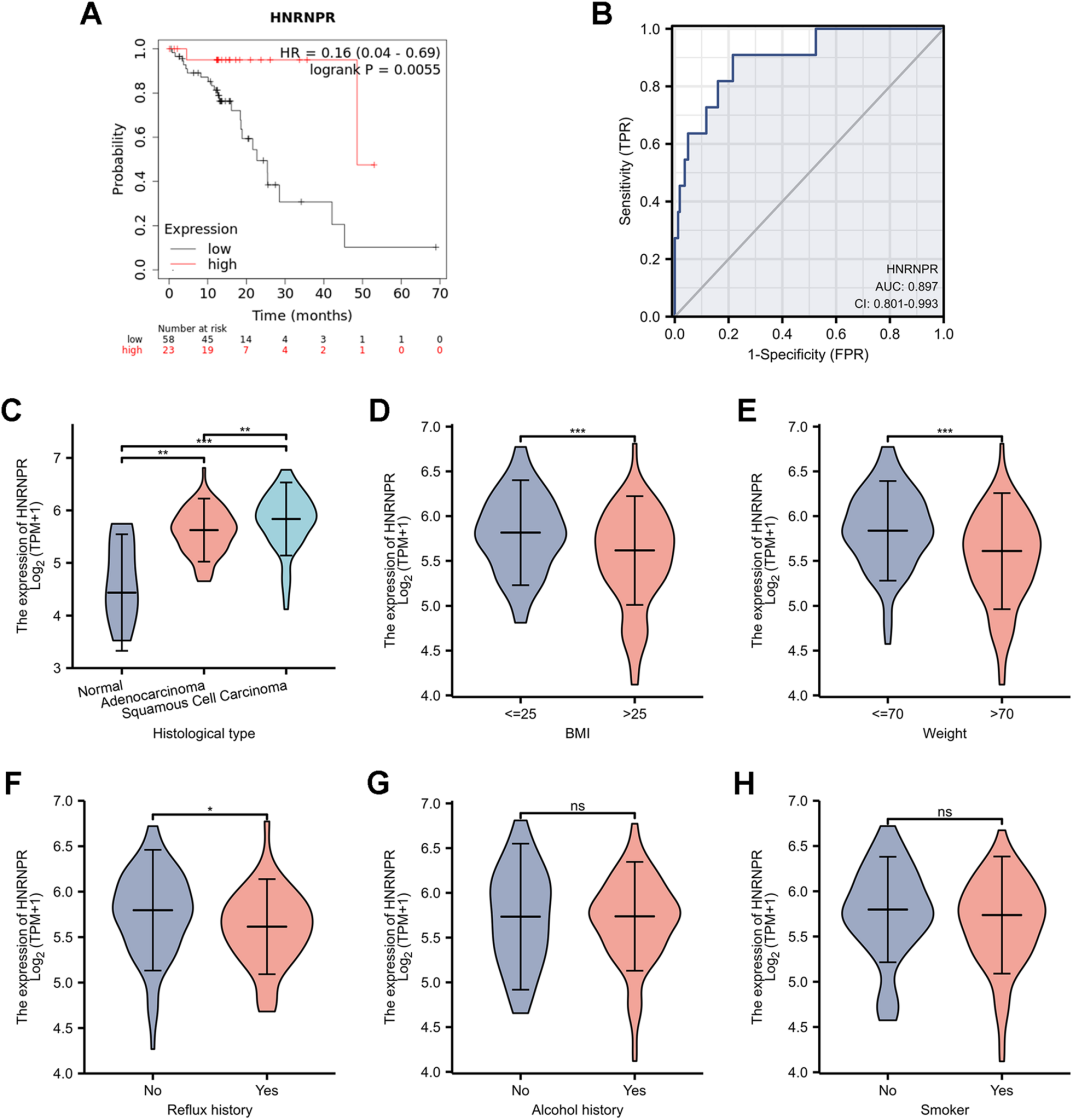
The relationship between the HNRNPR expression and clinicopathological parameters in ESCA patients. **A** Kaplan–Meier graph showing the survival analyses of ESCA patients based on HNRNPR expression. **B** ROC curve analysis of HNRNPR diagnosis in ESCA. The HNRNPR mRNA expression level was expressed in **C** histological type, **D** weight, **E** Body mass index (BMI), **F** reflux history, **G** smoking, and **H** alcohol consumption. *P < 0.05, **P < 0.01, ***P < 0.001

Additionally, Kaplan–Meier survival analysis suggested that patients whose tumors exhibited higher expression of HNRNPR had a decreased overall survival (Fig. 2A). The ROC curve analysis revealed that HNRNPR had high predictive accuracy, with a ROC curve of 0. 897 (95% CI:0. 801–0. 993) (Fig. 2B).

Additionally, we examined the significant clinicopathological role of HNRNPR in ESCA using the TCGA database, which included histological type, weight, Body mass index (BMI), reflux history, smoking, and alcohol intake. The results indicated that HNRNPR expression was significantly higher in ESCC than in EAC (Fig. 2C). HNRNPR mRNA expression was decreased in people with higher body weight and BMI (Fig. 2D, E). HNRNPR also affected whether the patient developed reflux or not (Fig. 2F). There was, however, no significant change in HNRNPR expression between patients who smoked and those who did not (Fig. 2G, H).

### Relationship between HNRNPR expression and PET metabolic parameters

**Fig. 3:**
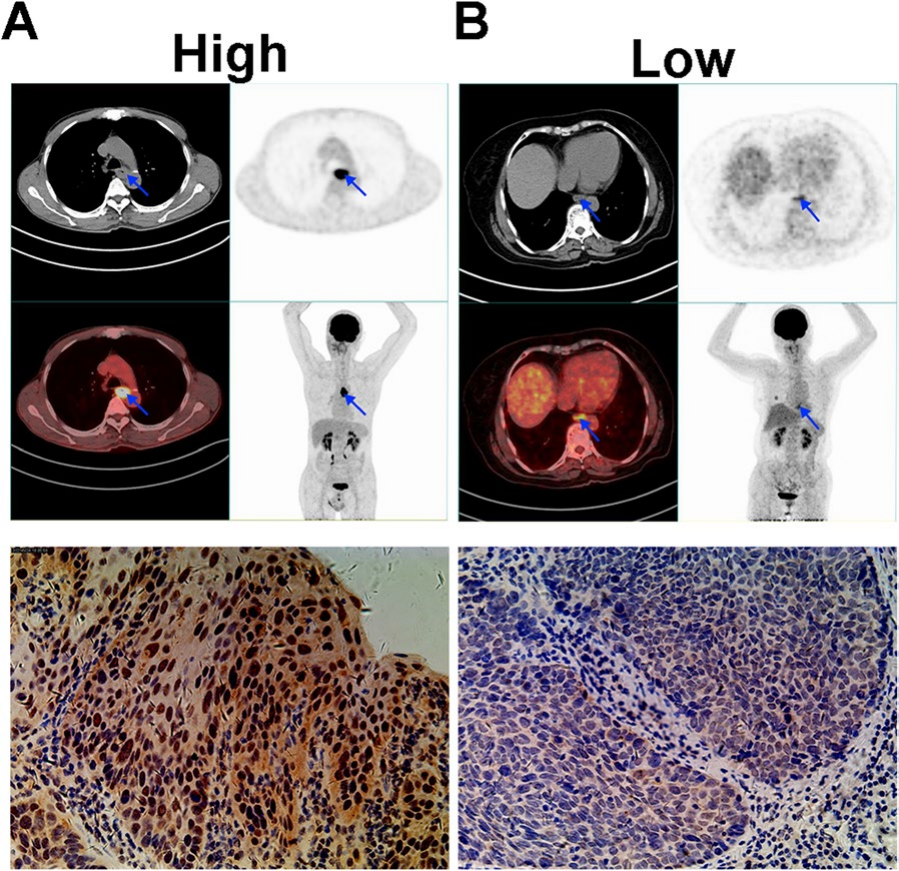
PET/CT imaging for ESCA patient with different SUV_max_, SUV_mean_, TLG and MTV. **A** SUV_max_ = 17.67, SUV_mean_ = 10.82, TLG = 72.29 and MTV = 7.15. **B** SUV_max_ = 5.64, SUV_mean_ = 3.09, TLG = 8.42, and MTV = 2.76

By comparing the PET parameter values of patients to corresponding IHC results, we discovered that FDG uptake rate also had an effect on HNRNPR expression (Fig. 3A, B). Notably, HNRNPR was expressed only in the cytoplasm or in both the nucleus and cytoplasm, which may be related to the state of the cells at the time of expression and the functions activated by HNRNPR [19, 26].

**Fig. 4:**
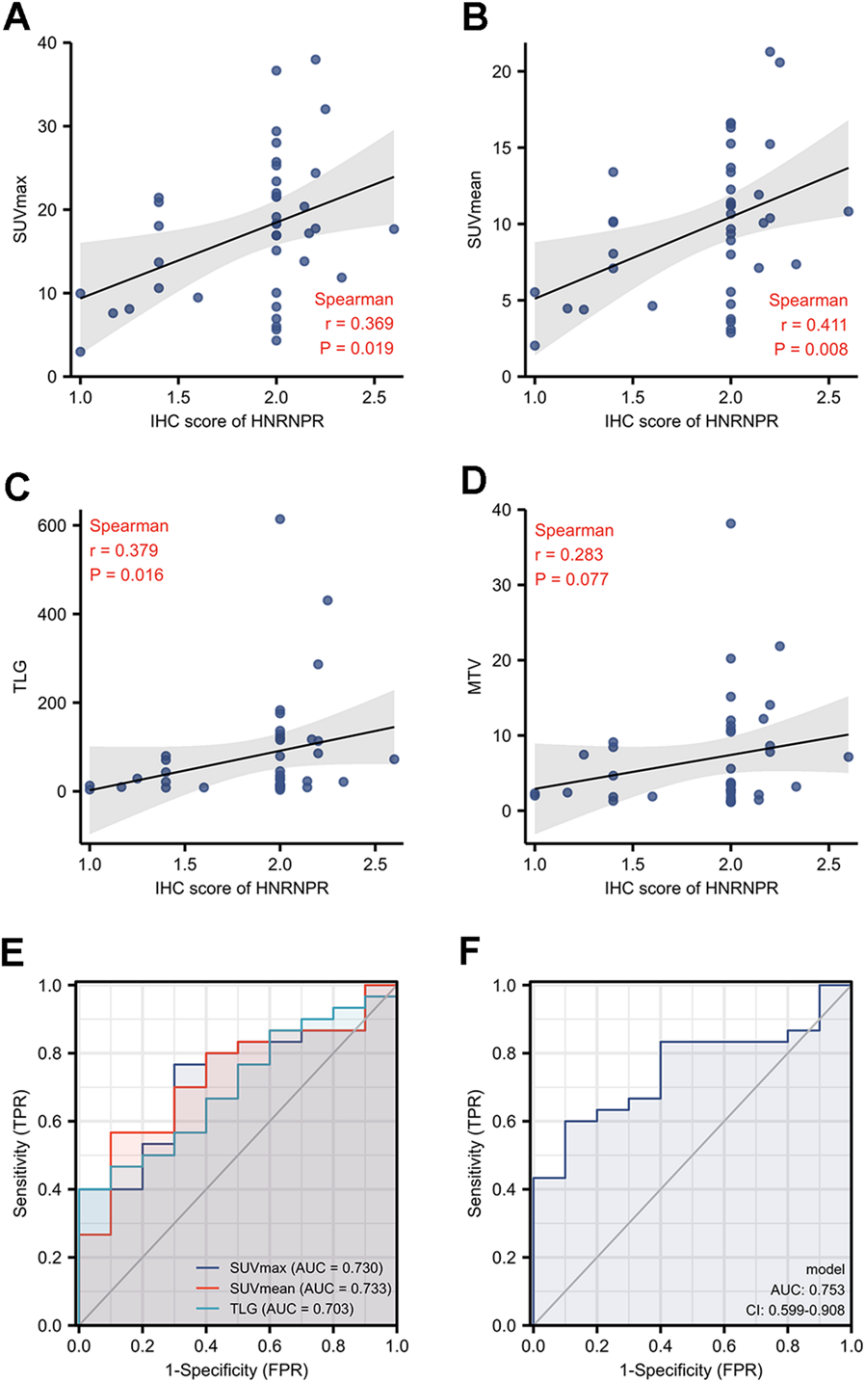
The correlation between the HNRNPR expression and PET parameters in ESCA. The correlation between HNRNPR IHC intensity levels and PET/CT metabolic parameters: SUV_max_ (**A**), SUV_mean_ (**B**), TLG (**C**), and MTV (**D**). The ROC curve was obtained by analyzing IHC scores and parameter (SUV_max_ and SUV_mean_ and TLG) values (**E**), using the combination of the three parameters (**F**)

To further investigate the relationship between ^18^F-FDG PET/CT metabolic parameters and HNRNPR expression in patients with ESCA, the IHC staining intensity of HNRNPR in tumor samples was determined, as well as the correlation with SUV_max_, SUV_mean_, TLG, and MTV. As shown in the figure (Fig. 4A–D), the HNRNPR score was positively correlated with SUV_max_, SUV_mean_, and TLG (rho = 0. 369, 0. 411, 0. 379, respectively, p < 0. 05), but not with MTV. We hypothesized that SUV_max_, SUV_mean_, and TLG may be used to predict HNRNPR expression levels in ESCA based on the above data.

Logistic analysis was used to determine the significance of each significant parameter in the HNRNPR expression. The area under the SUV_max_, SUV_mean_, and TLG ROC curves was 0. 730, 0. 733, 0. 703; and the combined index of the ROC curves for these three indexes was 0. 753, implying that the accuracy of the answer obtained by considering all three parameters comprehensively is more accurate (Fig. 4E, F).

### HNRNPR Co-expression networks indicate the potential function of HNRNPR in ESCA

**Fig. 5:**
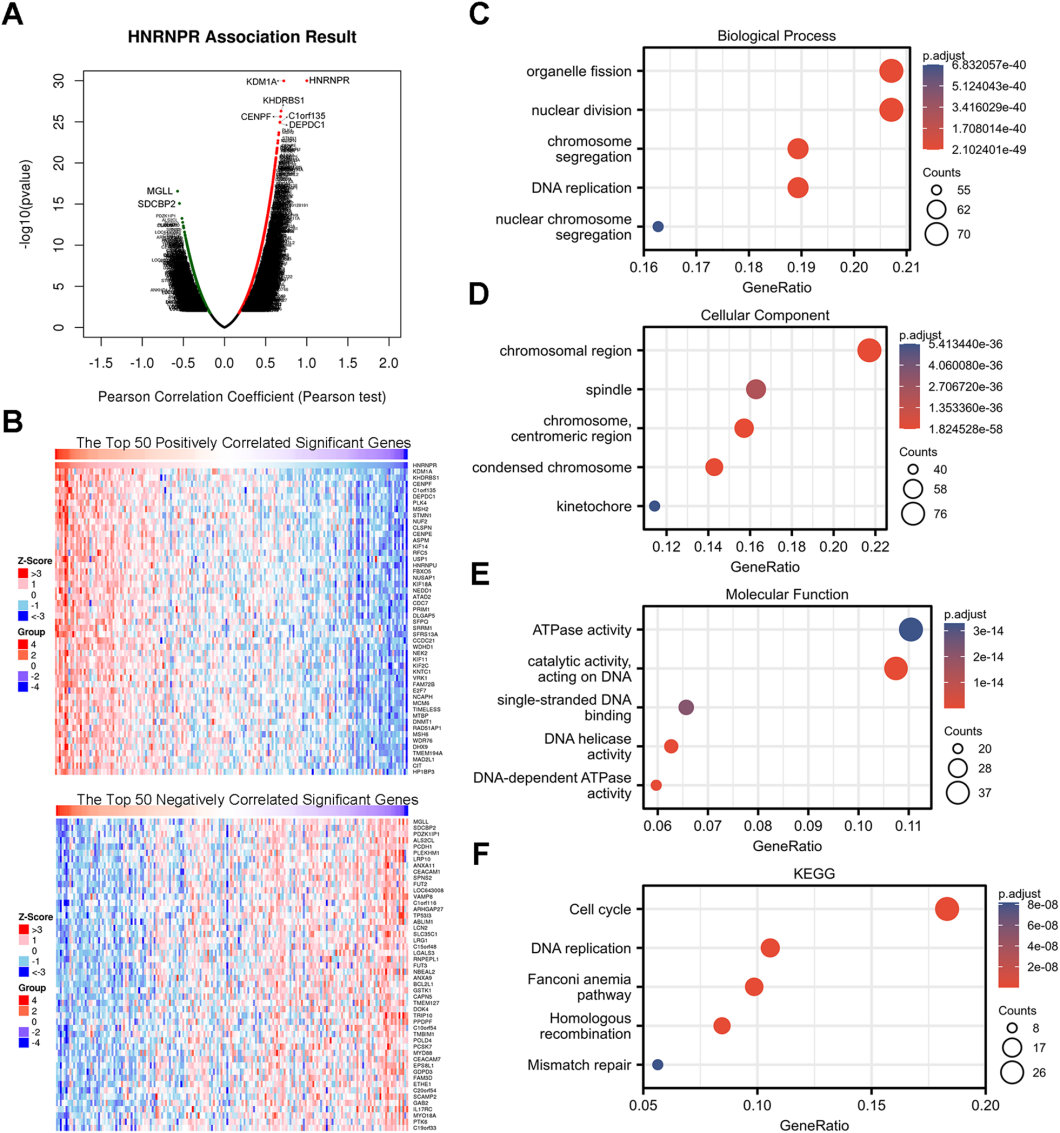
Enrichment analysis of HNRNPR gene co-expression network in ESCA. **A** Volcano map showing co-expression genes associated with HNRNPR expression in TCGA ESCA data sets. **B** Heat maps showing the top 50 co-expression genes that were positively and negatively correlated with HNRNPR expression in the ESCA data sets. **C**–**E** Enrichment analysis of gene ontology (GO) terms for HNRNPR co-expression genes. **F** Enrichment analysis of Kyoto Encyclopedia of Genes and Genomes (KEGG) terms for HNRNPR co-expression genes

There were 20,140 HNRNPR-related co-expressed genes in TCGA ESCA, and we performed gene enrichment analysis using the LinkedOmics database. As shown in Fig. 5A, 11827 genes were positively correlated with HNRNPR (red dot) while 8313 genes were negatively correlated (green dot). KDM1A (r = 0.720, p = 1.04E−30), KHDRBS1(r = 0.687, p = 4.88E−27), CENPF (r = 0.680, p = 2.30E−26), C1orf135(r = 0.674, p = 1.07E-25), DEPDC1(r = 0.673, p = 1.19E−25) had the highest positive correlation with HNRNPR. Notably, MGLL (r = − 0.571, p = 2.71E−17) and SDCBP2 (r = − 0.548, p = 8.36E−16) showed the strongest negative correlation with HNRNPR. A heat map showing the top 50 significant genes that were positively and negatively correlated with HNRNPR expression is shown in Fig. 5B. Among them, KDM1A and KHDRBS1 were previously found to be significantly related to the occurrence and prognosis of ESCA [27, 28].

The GO function and KEGG pathway enrichment analysis of the first 400 co-expressed genes positively correlated with HNRNPR expression were analyzed in line with the p < 0.05 and q < 0.25 as cut-off values. HNRNPR co-expressed genes were implicated in 18 KEGG and multiple GO functions, and the GO functions involved were classified as follows: 549 biological processes (GO-BP), 102 cell components (GO-CC), and 70 molecular functions (GO-MF). The bubble graph demonstrates the top 5 messages, respectively (Fig. 5C–F).

### Functions of HNRNPR and its related genes

**Fig. 6:**
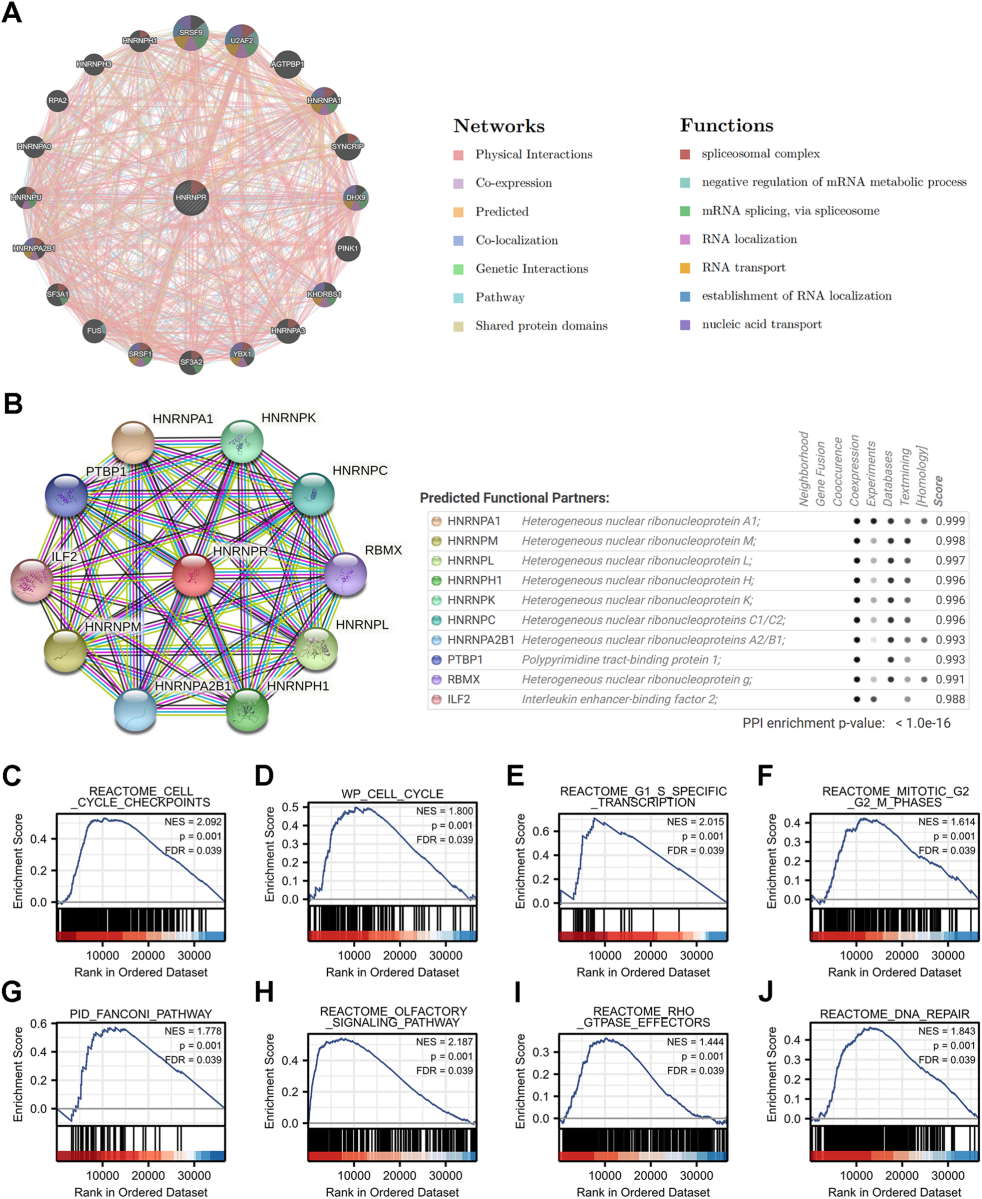
Analysis of GGI, PPI, and GSEA of HNRNPR gene. **A** GGI network of HNRNPR. **B** PPI interaction network of HNRNPR. **C**–**F** GSEA showed that high HNRNPR expression was positively correlated with pathways related to the cell cycle. Pathway enriched in the Fanconi pathway (**G**), Olfactory signaling pathway (**H**), RHO GTPase effectors (**I**), and DNA repair (**J**)

20 HNRNPR-related genes were analyzed in terms of function by the GeneMANIA database. They surrounded HNRNPR and formed the GGI network. Genes in the GGI network were intertwined to form many "intersection points", which represented physical interactions (77.64%), co-expression (8.01%), predicted (5.37%), co-localization (3.63%), genetic interactions (2.87%), pathway (1.88%), and Shared protein domains (0.60%). Five genes, including SRSF9 (serine and arginine-rich splicing factor 9), U2AF2 (U2 small nuclear RNA auxiliary factor 2), AGTPBP1 (ATP/GTP binding protein 1), HNRNPA1 (heterogeneous nuclear ribonucleoprotein A1), and SYNCRIP (synaptotagmin binding cytoplasmic RNA interacting protein), had the strongest correlation with HNRNPR. The strongest interaction among the 21 genes was physical contact. Most of these 21 genes were related to splicing, metabolism, and, mRNA transport (Fig. 6A).

STRING was used to further analyze the PPI network of HNRNPR. The genes associated with HNRNPR were HNRNPA1, HNRNPM, HNRNPL, HNRNPH1, HNRNPK, HNRNPC, HNRNPA2B1, PTBP1, RBMX, and ILF2. Their combined scores are shown in Fig. 6B.

We deduced from gene enrichment analysis that HNRNPR participates in and regulates the cell cycle in a variety of pathways (Fig. 6C–F). Figure 6G–J shows the list of several other pathways in which HNRNPR is involved, including the Fanconi pathway, which is a risk factor for ESCA.

### Correlation between HNRNPR Expression and m6A modification in ESCA

m6A methylation is a critical RNA modification in eukaryotic cells, affecting numerous aspects of RNA metabolism. It not only contributes to the occurrence and development of cancer but also provides new ideas for its early diagnosis and treatment. The PI3K–AKT–mTOR, RAS–MAPK, and/or MYC signaling pathways are the main signal pathways of m6A [23].

**Fig. 7:**
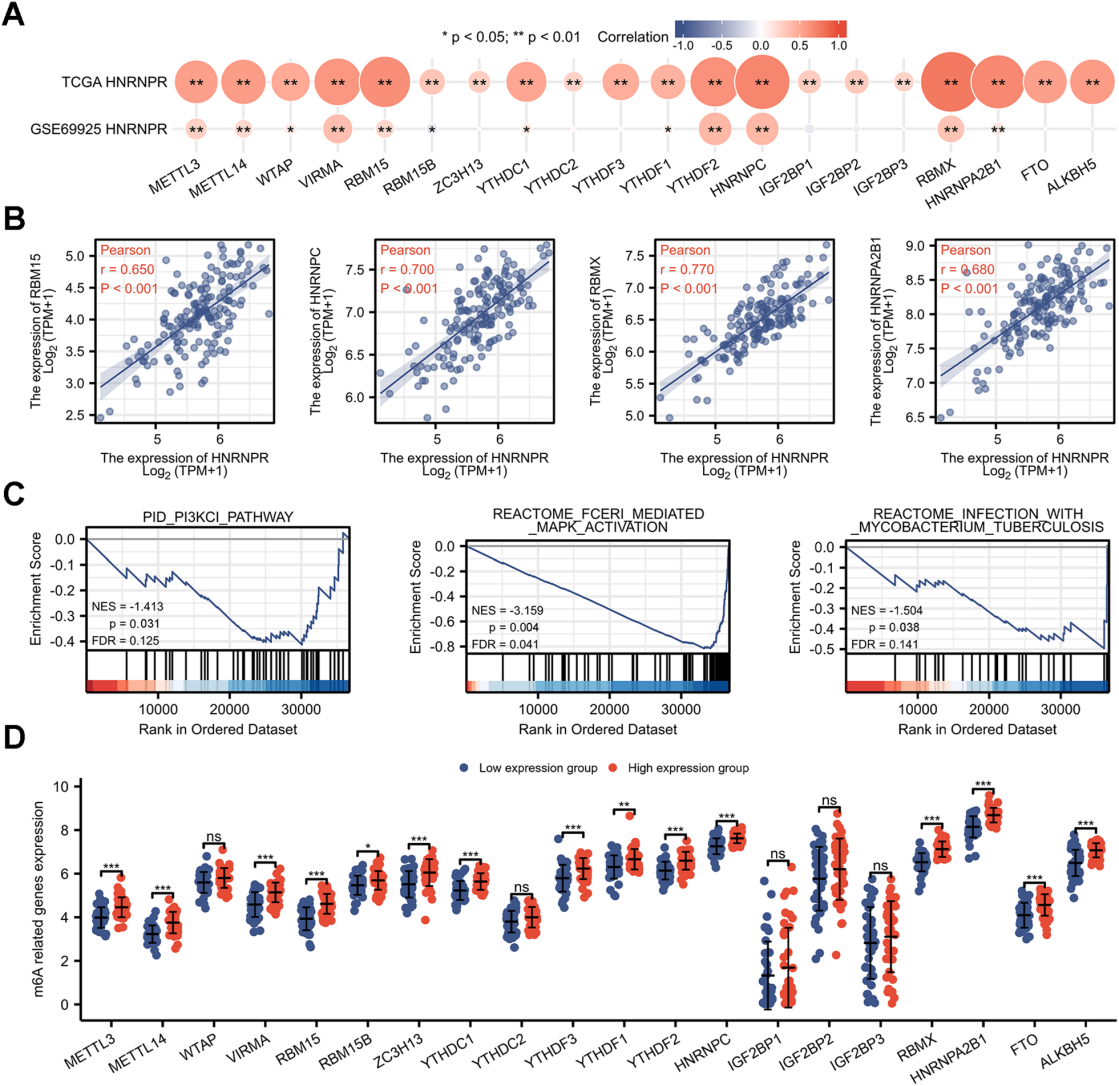
Correlation between HNRNPR expression and m6A-related genes in ESCA. **A** The correlation between m6A-related genes and HNRNPR was obtained by analyzing the TCGA database and GSE69925 dataset. **B** Scatter plot of the four highly correlated genes, including RBM15, HNRNPC, RBMX, and HNRNPA2B1. **C** HNRNPR participates in the m6A-related pathways. **D** The difference between m6A-related gene expression in the high and low HNRNPR expression groups in ESCA patients. *P < 0.05, **P < 0.01, ***P < 0.001

According to the analysis of the TCGA dataset, 20 m6A-related genes were positively correlated with the expression of HNRNPR (P < 0.05), whereas in the GEO dataset, the HNRNPR expression is positively correlated with Methyltransferase 3 (METTL3), Methyltransferase 14 (METTL14), WT1 Associated Protein (WTAP), Vir Like m6A Methyltransferase Associated (VIRMA), RNA Binding Motif Protein 15 (RBM15), YTH Domain Containing 1 (YTHDC1), YTH N6-Methyladenosine RNA Binding Protein 1 (YTHDF1), YTH N6-Methyladenosine RNA Binding Protein 2 (YTHDF2), HNRNPC, RBMX, HNRNPA2B1; and negatively correlated with RBM15B (Fig. 7A, B). As shown in Fig. 7C, HNRNPR is involved in a number of the pathways outlined previously. There is differential expression in many m6A-related genes between high and low groups with HNRNPR expression in ESCA (Fig. 7D).

### Correlations between HNRNPR expression and glycolysis in ESCA

**Fig. 8:**
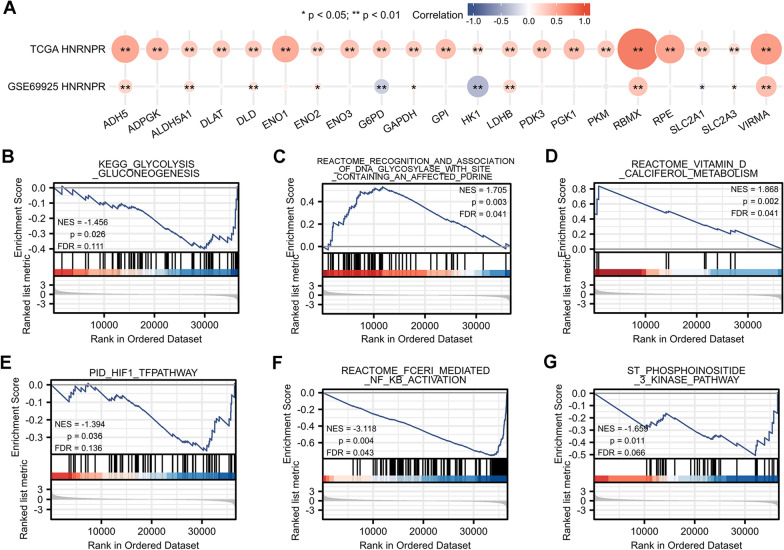
Correlation between HNRNPR expression and glycolysis-related genes in ESCA. **A** The correlation between glycolysis-related genes and HNRNPR in the TCGA database and GSE69925 dataset. **B**–**G** HNRNPR was involved in glycolysis-related pathways. *P < 0.05, **P < 0.01

The correlation between HNRNPR and glycolysis-related genes was analyzed using the TCGA and GEO databases. As shown in Fig. 8A, HNRNPR had a strong correlation with ADH5 (Alcohol Dehydrogenase 5), ALDH5A1 (Aldehyde Dehydrogenase 5 Family Member A1), DLD (Dihydrolipoamide Dehydrogenase), ENO2 (Enolase 2), G6PD (Glucose-6-Phosphate Dehydrogenase), GAPDH (Glyceraldehyde-3-Phosphate Dehydrogenase), HK1 (Hexokinase 1), LDHB (Lactate Dehydrogenase B), RBMX (RNA Binding Motif Protein X-Linked), SLC2A1 (Solute Carrier Family 2 Member 1), SLC2A3 (Solute Carrier Family 2 Member 3), and VIRMA. Additionally, except for the GSE69925 data set, G6PD, HK1, and SLC2A1 exhibited a negative correlation, whereas the rest showed a positive correlation.

Fig. 8B–G shows that HNRNPR was involved in many glycolysis-related pathways.

## Discussion

In 2020, ESCA accounted for one in every 18 cancer-related deaths, and the incidence has been increasing in some countries in recent years [1, 29]. Despite constant advancements in medical technology, there is still no reliable biomarker for the early diagnosis of ESCA. This study primarily discusses the expression and role of HNRNPR in ESCA, its potential as a biomarker of ESCA, with a particular emphasis on the relationship between HNRNPR expression and PET-related parameters, which not only provides an effective method for early screening of ESCA but also enriches the diagnosis and treatment strategies for ESCA, as well as facilitates effective prognosis evaluation to improve the survival rate of patients.

HNRNPR is a member of the hnRNPs family of proteins, which have been designated as the ‘‘core’’ hnRNP proteins and are increasingly recognized as multi-functional [30, 31]. Increasing evidence indicates that HNRNPR and its family have garnered considerable interest and have been identified as cancer biomarkers [32]. While HNRNPR has not been extensively investigated in cancer, the significance of its co-family genes in a variety of cancers has been gradually discovered. For example, HNRNPK has been shown to be a therapeutic target for cholangiocarcinoma [33] and cervical cancer [34]; the expression of HNRNPI [35] has been shown to influence the development of colitis into colorectal cancer; and in liver cancer, HNRNPA1 [36] has been shown to affect patient prognosis, while HNRNPAB [37] promotes metastasis. Additionally, research of the Oncomine online database and the TCGA dataset revealed that HNRNPR expression was significantly increased in the majority of pan-cancers, including ESCA. As a hnRNPs homologous gene, HNRNPR is structurally and functionally comparable to other members of this family and considered to have the ability to act as a proto-oncogene in numerous cancers.

Results of immunohistochemistry revealed that HNRNPR was present in the nucleus and cytoplasm, but the binding sites of HNRNPR in the nucleus and cytoplasm were different, implying that HNRNPR plays distinct roles depending on its localization [19, 38]. Previous research has discovered that, in addition to the most common nucleus, hnRNPs are also present in stress granules in the cytoplasm when cells are exposed to oxidative insult. Not only do stress particles regulate the stress response, but they also regulate virus infection, signaling pathways, and abnormal formation of stress particles, which can result in a variety of diseases, including cancer [18, 39]. Other studies have established that the HNRNPR protein is also a component of stress granules and that it has an effect on their formation and decomposition [18]. The above results suggest that the expression position of HNRNPR can reflect the state of cells. The role of HNRNPR in tumors in the context of stress particles need to be clarified. All of these must be confirmed through more experiments.

PPI plays an important role in regulating most biological mechanisms, and its imbalance results in a variety of diseases, including cancer [40]. In this experiment, through the STRING, there is PPI between HNRNPR and PTBP1, RBMX and ILF2 in addition to proteins from the same family. Among these, ILF2 [41] has been shown to affect the metabolic adaptation of ESCC. PPI is also one of the factors promoting the formation of stress granules mentioned previously. The interaction between HNRNPR and a variety of proteins indicates that HNRNPR may have the ability to act like other cancer proto-oncogenes. Additionally, immunoprecipitation coupled with mass spectrometry analysis was used to validate the interaction between HNRNPR and SOX2 [42]. SOX2 plays a critical role in the control of embryonic cells and various adult stem cell populations and is used as a proto-oncogene [42–44]. It has several effects on the proliferation, migration, invasion, and metastasis of cancer cells, and plays a significant role in the development and treatment of cancer [43, 44]. SOX2 has been shown to be expressed in different in cancers, but its expression in ESCC has been not been well-studied. It can regulate invasiveness and differentiation of ESCC cells, and the prognosis of ESCC patients after surgical resection [43, 45, 46]. Although no corresponding experiments were carried out in this study, it can be inferred that HNRNPR may affect the occurrence and development of ESCA through protein–protein interactions with SOX2, which further confirms the potential of HNPNPR as a biomarker of ESCA.

m6A is the most abundant methylation modification which modulates the occurrence and development of cancer by regulating biological function [23]. In this study, there was a strong correlation between HNRNPR and 20 m6A-related genes, and HNRNPRR expression also affected the expression of these genes in ESCA. These findings show that HNRNPR may be implicated in the regulation of methylation in ESCA which is dominated by m6A. FTO [47], RBMX [48], METTL3 [5], and METTL14 [49, 50], have been demonstrated to be tumor proto-oncogenes or biomarkers through m6A, with METTL3 serving as a pathological diagnostic index and potential therapeutic target for ESCA [5], and with RBMX encoding HNRNPG to affect m6A, also a member of the hnRNPs family [51]. Therefore, we hypothesize that HNRNPR may promote ESCA by modifying m6A-related genes, hence affecting the level of tumor methylation, and eventually resulting in ESCA progression.

Enrichment analysis revealed that HNRNPR is involved in a variety of pathways regulating the cell cycle, thus affecting cell division, translation, and so on. As is well known, cell cycle dysregulation is a common hallmark of human cancer, and numerous cancer treatments are based on the cell cycle [52–54]. Additionally, there were experiments demonstrated that overexpression of HNRNPR in GC significantly accelerated the process of cell cycle and promoted tumor cell proliferation and invasion, indicating that HNRNPR works as a pro-oncogene in the occurrence and development of GC [20]. Gastric and esophageal cancers share numerous similarities in terms of histological types, risk factors, etc., as both are upper digestive tract tumors. Additionally, HNRNPR is overexpressed in ESCA. From the above information, it may be deduced that HNRNPR is a proto-oncogene in ESCA.

ESCA is associated with a variety of clinical factors, among which BMI is the most studied. Epidemiological studies have shown that body mass index (BMI) and weight gain are associated with a decreased risk of ESCA [55, 56]. In this study, HNRNPR expression was significantly higher in patients with BMI < 25 or weight less than 70 kg compared to patients with BMI > 25 or weight more than 70 kg, which was consistent with the epidemiological investigation. The above appears to imply a relationship between HNRNPR expression and obesity. It is hypothesized that when weight gain and BMI increase, HNRNPR expression is suppressed, reducing the risk of ESCA. Whether this provides an explanation for this phenomenon at the genetic level remains to be further explored. Fanconi Anemia has been associated with an increased risk of ESCA, and GASE analysis revealed that HNRNPR may participate in the Fannie anemia pathway, which complements the important role of HNRNPR in ESCA [55, 57]. Additionally, in terms of prognosis, survival rate analysis and the ROC curve analysis both support the value of HNRNPR in ESCA. These clinical indicators show that HNRNPR has clinical use as an ESCA biomarker.

^18^F-FDG PET/CT takes advantage of the fact that cancer cells have a higher glucose metabolic rate than normal cells to accurately reflect the glucose metabolism and burden of tumor cells in vivo. ^18^F-FDG is a glucose analogue, and up-regulation of glycolysis can lead to an increase in ^18^F-FDG uptake, indicating that quantitative imaging with ^18^F-FDG PET/CT can reflect the glucose metabolism and burden of tumor cells in vivo [8, 9]. Previous research has established that ^18^F-FDG PET/CT quantitative assessment of tumor metabolism is an effective and clinically useful technique [7]. In the current clinical application for ESCA, ^18^F-FDG PET/CT has been routinely examined for tumor staging, which can aid in the initial staging and can assist in the evaluation of distant metastasis and prediction of treatment response [58, 59]. Additionally, PET parameters have been shown to be predictive and prognostic markers for ESCA [16]. In this study, HNRNPR expression was associated with PET for the first time. The correlation analysis of IHC score and PET-related parameters in patients with ESCA revealed a strong correlation between HNRNPR expression and SUV_max_, SUV_mean,_ and TLG in ESCA. When combined with GESA analysis, it is clear that HNRNPR participates in glycolysis in a variety of ways and has a strong association with a large number of glycolysis-related genes. We hypothesize that HNRNPR may promote the occurrence and development of ESCA by enhancing glycolysis in ESCA tissue. This result not only confirms to previous findings, but also suggests that TLG can be used as a biomarker, and HNRNPR can be used as a biomarker for ESCA. Additionally, analysis of the clinical ROC curve showed that the accuracy of diagnosis was improved by comprehensively considering the detection results of many PET parameters. However, the results above are limited by the small sample size, and additional experiments required to confirm the findings.

There are some limitations in this study. Since the sample size collected in the retrospective study is relatively small, the results obtained should be carefully interpreted. Future studies should enroll large samples to verify our conclusions.

## Conclusion

In conclusion, HNRNPR overexpression affects not only the clinical manifestations of ESCA patients but also their malignant degree and clinical prognosis. Additionally, HNRNPR expression correlates with ^18^F-FDG uptake. ^18^F-FDG PET/CT can predict HNRNPR expression in ESCA, and it is positively correlated with SUV_max_, SUV_mean_, and MTV. HNRNPR exerts a strong regulatory effect on DNA replication and RNA transcription, as well as on cell cycle regulation. Additionally, HNRNPR is involved in glycolysis and participates in a variety of glycolysis pathways. These findings imply that HNRNPR may influence ^18^F-FDG uptake by controlling glycolysis and the physiological activities of tumor cells by regulating the cell cycle and glycolysis. Therefore, HNRNPR can be used as a molecular marker and therapeutic target for ESCA and can be combined with ^18^F-FDG PET/CT to provide a non-invasive, simple, and more accurate examination for ESCA.

## Data Availability

The datasets presented in this study can be found in online repositories. The data used to support the findings of this study are included within the article.

## Abbreviations

HNRNPR: Heterogeneous nuclear ribonucleoprotein R
ESCA: Esophageal carcinoma
^18^F-FDG: ^18^F-fluorodeoxyglucose
PET/CT: Positron emission tomography/computerized tomography scan
AC: Squamous cell carcinoma (SCC) and adenocarcinoma
hnRNPs: Heterogeneous nuclear ribonucleoproteins
GC: Gastric cancer
m6A: N6-methyladenosine
RT-PCR: Real-time polymerase chain reaction
DAB: Diaminobenzaldehyde
GSEA: Gene Set Enrichment Analysis
BMI: Body mass index
SUV_max_: Maximum standardised uptake value
SUV_mean_: Mean standardised uptake value
TLG: Total lesion glycolysis
MTV: Metabolic tumour volume
GO: Gene ontology
KEGG: Kyoto Encyclopedia of Genes and Genomes
BP: Biological processes
CC: Cell components
MF: 70 Molecular functions
SRSF9: Serine and arginine-rich splicing factor 9
U2AF2: U2 small nuclear RNA auxiliary factor 2
AGTPBP1: ATP/GTP binding protein 1
HNRNPA1: Heterogeneous nuclear ribonucleoprotein A1
SYNCRIP: Synaptotagmin binding cytoplasmic RNA interacting protein
HNRNPA1: Heterogeneous Nuclear Ribonucleoprotein A1
HNRNPM: Heterogeneous Nuclear Ribonucleoprotein M
HNRNPL: Heterogeneous Nuclear Ribonucleoprotein L
HNRNPH1: Heterogeneous Nuclear Ribonucleoprotein H1
HNRNPK: Heterogeneous Nuclear Ribonucleoprotein K
HNRNPC: Heterogeneous Nuclear Ribonucleoprotein C
HNRNPA2B1: Heterogeneous Nuclear Ribonucleoprotein A2/B1
PTBP1: Polypyrimidine Tract Binding Protein 1
RBMX: RNA Binding Motif Protein X-Linked
HNRNPF: Heterogeneous Nuclear Ribonucleoprotein F
ILF2: Interleukin Enhancer Binding Factor 2
METTL3: Methyltransferase 3
METTL14: Methyltransferase 14
WTAP: WT1 Associated Protein
VIRMA: Vir Like m6A Methyltransferase Associated
RBM15: RNA Binding Motif Protein 15
YTHDC1: YTH Domain Containing 1
YTHDF1: YTH N6-Methyladenosine RNA Binding Protein 1
YTHDF2: YTH N6-Methyladenosine RNA Binding Protein 2
ADH5: Alcohol Dehydrogenase 5
ALDH5A1: Aldehyde Dehydrogenase 5 Family Member A1
DLD: Dihydrolipoamide Dehydrogenase
ENO2: Enolase 2
G6PD: Glucose-6-Phosphate Dehydrogenase
GAPDH: Glyceraldehyde-3-Phosphate Dehydrogenase
HK1: Hexokinase 1
LDHB: Lactate Dehydrogenase B
SLC2A1: Solute Carrier Family 2 Member 1
SLC2A3: Solute Carrier Family 2 Member 3

## References

[1] Sung H (2021). Global cancer statistics 2020: GLOBOCAN estimates of incidence and mortality worldwide for 36 cancers in 185 Countries. CA Cancer J Clin.

[2] Bray F (2018). Global cancer statistics 2018: GLOBOCAN estimates of incidence and mortality worldwide for 36 cancers in 185 countries. CA Cancer J Clin.

[3] Lu Y, Guo L, Ding G (2019). PD1+ tumor associated macrophages predict poor prognosis of locally advanced esophageal squamous cell carcinoma. Future Oncol.

[4] Chen Y (2019). The clinical outcomes of locally advanced cervical esophageal squamous cell carcinoma patients receiving curative concurrent chemoradiotherapy: a population-based propensity score-matched analysis. Cancers.

[5] Liu X (2020). Overexpression of METTL3 associated with the metabolic status on 18F-FDG PET/CT in patients with esophageal carcinoma. J Cancer.

[6] Kamangar F, Dores GM, Anderson WF (2006). Patterns of cancer incidence, mortality, and prevalence across five continents: defining priorities to reduce cancer disparities in different geographic regions of the world. J Clin Oncol.

[7] Ott K (2006). Metabolic imaging predicts response, survival, and recurrence in adenocarcinomas of the esophagogastric junction. J Clin Oncol.

[8] Nair VS (2012). Prognostic PET 18F-FDG uptake imaging features are associated with major oncogenomic alterations in patients with resected non-small cell lung cancer. Can Res.

[9] Kawada K (2012). Relationship between18F-fluorodeoxyglucose accumulation and KRAS/BRAF mutations in colorectal cancer. Clin Cancer Res.

[10] Koppenol WH, Bounds PL, Dang CV (2011). Otto Warburg's contributions to current concepts of cancer metabolism. Nat Rev Cancer.

[11] Wang S (2019). Circular RNA FOXP1 promotes tumor progression and Warburg effect in gallbladder cancer by regulating PKLR expression. Mol Cancer.

[12] Mathupala SP, Ko YH, Pedersen PL (2009). Hexokinase-2 bound to mitochondria: cancer's stygian link to the “Warburg effect” and a pivotal target for effective therapy. Semin Cancer Biol.

[13] Levine EA (2006). Predictive value of 18-fluoro-deoxy-glucose-positron emission tomography (18F-FDG-PET) in the identification of responders to chemoradiation therapy for the treatment of locally advanced esophageal cancer. Ann Surg.

[14] Blackstock AW (2006). A prospective evaluation of the impact of 18-F-fluoro-deoxy-D-glucose positron emission tomography staging on survival for patients with locally advanced esophageal cancer. Int J Radiat Oncol Biol Phys.

[15] Monjazeb AM (2010). Outcomes of patients with esophageal cancer staged with [(1)(8)F]fluorodeoxyglucose positron emission tomography (FDG-PET): can postchemoradiotherapy FDG-PET predict the utility of resection?. J Clin Oncol.

[16] Goodman KA (2021). Randomized phase II study of PET response-adapted combined modality therapy for esophageal cancer: mature results of the CALGB 80803 (Alliance) trial. J Clin Oncol.

[17] Choi YD (1986). Heterogeneous nuclear ribonucleoproteins: role in RNA splicing. Science.

[18] Duijkers FA (2019). HNRNPR variants that impair homeobox gene expression drive developmental disorders in humans. Am J Hum Genet.

[19] Ghanawi H (2021). Loss of full-length hnRNP R isoform impairs DNA damage response in motoneurons by inhibiting Yb1 recruitment to chromatin. Nucleic Acids Res.

[20] Chen E (2019). HnRNPR-CCNB1/CENPF axis contributes to gastric cancer proliferation and metastasis. Aging.

[21] Yang J (2021). 18F-FDG PET/CT metabolic parameters correlate with EIF2S2 expression status in colorectal cancer. J Cancer.

[22] Li Y (2019). Molecular characterization and clinical relevance of m(6)A regulators across 33 cancer types. Mol Cancer.

[23] Fabbri L (2021). The plasticity of mRNA translation during cancer progression and therapy resistance. Nat Rev Cancer.

[24] Rapino F (2018). Codon-specific translation reprogramming promotes resistance to targeted therapy. Nature.

[25] Chang CH (2013). Posttranscriptional control of T cell effector function by aerobic glycolysis. Cell.

[26] Pinol-Roma S, Dreyfuss G (1993). hnRNP proteins: localization and transport between the nucleus and the cytoplasm. Trends Cell Biol.

[27] Han J (2021). Lysine-specific histone demethylase 1 promotes oncogenesis of the esophageal squamous cell carcinoma by upregulating DUSP4. Biochemistry.

[28] Wang Y (2015). Sam68 promotes cellular proliferation and predicts poor prognosis in esophageal squamous cell carcinoma. Tumour Biol.

[29] Demeester SR (2009). Epidemiology and biology of esophageal cancer. Gastrointest Cancer Res.

[30] Beyer AL (1977). Identification and characterization of the packaging proteins of core 40S hnRNP particles. Cell.

[31] Chaudhury A, Chander P, Howe PH (2010). Heterogeneous nuclear ribonucleoproteins (hnRNPs) in cellular processes: focus on hnRNP E1's multifunctional regulatory roles. RNA.

[32] Kudinov AE (2017). Musashi RNA-binding proteins as cancer drivers and novel therapeutic targets. Clin Cancer Res.

[33] Phoomak C (2019). O-GlcNAc-induced nuclear translocation of hnRNP-K is associated with progression and metastasis of cholangiocarcinoma. Mol Oncol.

[34] Zhang L (2016). Nujiangexathone A, a novel compound from *Garcinia nujiangensis*, suppresses cervical cancer growth by targeting hnRNPK. Cancer Lett.

[35] Jin Z (2017). hnRNP I regulates neonatal immune adaptation and prevents colitis and colorectal cancer. PLoS Genet.

[36] Zhou ZJ (2013). Overexpression of HnRNP A1 promotes tumor invasion through regulating CD44v6 and indicates poor prognosis for hepatocellular carcinoma. Int J Cancer.

[37] Zhou ZJ (2014). HNRNPAB induces epithelial-mesenchymal transition and promotes metastasis of hepatocellular carcinoma by transcriptionally activating SNAIL. Cancer Res.

[38] Briese M (2018). hnRNP R and its main interactor, the noncoding RNA 7SK, coregulate the axonal transcriptome of motoneurons. Proc Natl Acad Sci U S A.

[39] Protter D, Parker R (2016). Principles and Properties of Stress Granules. Trends Cell Biol.

[40] Rodrigues C, Pires D, Ascher DB (2021). mmCSM-PPI: predicting the effects of multiple point mutations on protein-protein interactions. Nucleic Acids Res.

[41] Zang B (2021). Metabolomic characterization reveals ILF2 and ILF3 affected metabolic adaptions in esophageal squamous cell carcinoma. Front Mol Biosci.

[42] Fang X (2011). Landscape of the SOX2 protein-protein interactome. Proteomics.

[43] Novak D (2020). SOX2 in development and cancer biology. Semin Cancer Biol.

[44] Chaudhary S (2019). Sox2: a regulatory factor in tumorigenesis and metastasis. Curr Protein Pept Sci.

[45] Bass AJ (2009). SOX2 is an amplified lineage-survival oncogene in lung and esophageal squamous cell carcinomas. Nat Genet.

[46] Saigusa S (2009). Correlation of CD133, OCT4, and SOX2 in rectal cancer and their association with distant recurrence after chemoradiotherapy. Ann Surg Oncol.

[47] Li Z (2017). FTO plays an oncogenic role in acute myeloid leukemia as a N(6)-methyladenosine RNA demethylase. Cancer Cell.

[48] Song Y (2020). RBMX contributes to hepatocellular carcinoma progression and sorafenib resistance by specifically binding and stabilizing BLACAT1. Am J Cancer Res.

[49] Shen D (2022). METTL14-mediated Lnc-LSG1 m6A modification inhibits clear cell renal cell carcinoma metastasis via regulating ESRP2 ubiquitination. Mol Ther Nucleic Acids.

[50] Dong L (2021). The loss of RNA N(6)-adenosine methyltransferase Mettl14 in tumor-associated macrophages promotes CD8(+) T cell dysfunction and tumor growth. Cancer Cell.

[51] Liu N (2017). N6-methyladenosine alters RNA structure to regulate binding of a low-complexity protein. Nucleic Acids Res.

[52] Malumbres M (2014). Cyclin-dependent kinases. Genome Biol.

[53] Manchado E, Guillamot M, Malumbres M (2012). Killing cells by targeting mitosis. Cell Death Differ.

[54] Hanahan D, Weinberg RA (2011). Hallmarks of cancer: the next generation. Cell.

[55] Abnet CC, Arnold M, Wei WQ (2018). Epidemiology of esophageal squamous cell carcinoma. Gastroenterology.

[56] Lahmann PH (2012). Body mass index, long-term weight change, and esophageal squamous cell carcinoma: is the inverse association modified by smoking status?. Cancer.

[57] Rosenberg PS, Alter BP, Ebell W (2008). Cancer risks in Fanconi anemia: findings from the German Fanconi Anemia Registry. Haematologica.

[58] Arnett A (2017). Utility of (18)F-FDG PET for predicting histopathologic response in esophageal carcinoma following chemoradiation. J Thorac Oncol.

[59] Cuellar SLB (2014). Clinical staging of patients with early esophageal adenocarcinoma: does FDG-PET/CT have a role?. J Thorac Oncol.

